# Veterinary Education

**Published:** 1900-12

**Authors:** 


### EDITORIALS.

### VETERINARY EDUCATION.

UNDER the heading "Correspondence" we publish in this issue a communication in relation to the needs and tendencies of veterinary education. There is no reason, with the experience of one hundred and thirty-eight years since the founding of the first veterinary school to guide us, why we should repeat the errors that have been made. There is no reason why veterinary schools in this country should have their development retarded and their usefulness impaired by conditions that were found in Europe a century ago to have a stunting and unwholesome effect. It is now time to realize, as our correspondent says, that the successful veterinary school is not a branch of a medical school, and that it should be a school of veterinary science rather than a school of veterinary medicine.

No school can furnish a complete education in any science or art. All that schools can do for students is to teach principles, so much detail as is necessary to the proper understanding and application of these principles, and then show the student how to apply his knowledge and how and where he may gain more. This additional learning, that is built upon the foundation the school has laid, and in the direction the school has pointed out, is the true education; it is this that is most useful to mankind.

It is evident that our Yankee friend is strongly of the opinion that the direction of veterinary education should be more to the animal husbandry side and less toward medicine. He states his case strongly, and there is much to say in favor of his view. But still it should be remembered that the veterinarian must always be an expert on the diseases of animals, and that this is his most important field. It is here that his greatest work has been accomplished and that his greatest usefulness lies. It is undeniably true, however, that many veterinarians are not in a position to render as useful service as they should, for the reason that the foundation of their technical training was laid within too narrow lines and without sufficient inclination toward practical animal husbandry.

While veterinarians cannot be too well trained in comparative medicine, they should be taught by men who are versed in comparative medicine in the broadest sense, and who understand how the knowledge they possess can best be applied to veterinary problems, and not by men who have only the narrower training of physicians.

Now and then a veterinary school is so fortunate as to have as a member of its Faculty a physician who has a real interest in the work of his veterinary classes, and who not only devotes much thought and time to them, but also makes material contributions to the general knowledge of his subject. Unfortunately, such men are rare—so, broadly speaking, for development in the veterinary sciences we must depend on veterinarians. Does not this mean that the teaching of veterinary students should, so far as possible, be entrusted to veterinarians?

If the union of veterinary and medical schools is logical, then the union of the public administrative work of the veterinarian and the physician is logical. If veterinary and medical schools should be attached to each other, then public administrative work along veterinary lines should be under the jurisdiction of boards of health. Cattle commissions, livestock sanitary boards, State veterinarians, and the Bureau of Animal Industry should then be under or associated with health boards instead of departments of agriculture. But this management would not be satisfactory nor successful, for proof of which witness the organization of such work in all States and countries.

The veterinary schools of France are under the Ministry of War; those of Germany are under the Ministry of Agriculture. The medical schools of these countries are under the Ministry of Education. The veterinary school in London is supported in part by the Royal Agricultural Society, and those of Scotland are under the patronage of the Highland and Agricultural Society.

There can be no doubt that the successful *and useful* veterinarians are, first, lovers and connoisseurs of animals; and, second, veterinary physiologists, pathologists, therapeutists, etc. The knowledge of and feeling for animals is an essential preliminary, and should be trained and developed as much as possible throughout the entire veterinary course. Hence, the equipment and teaching staff of a veterinary school must be much more than of a mere school of comparative medicine, and must embrace facilities for teaching the principles and practice of animal husbandry.

It must not be inferred, because the work of the veterinarian touches that of the physician here and there, that the veterinarian is exclusively a comparative pathologist, and that his work is a subordinate kind of medical practice. Should this view obtain, it were time to divorce veterinary science from the thraldom of the medical schools.

As to the situation at Harvard, we sincerely hope that the veterinary school may gain the support it deserves and reorganize on broad, modern lines.

